# Traumatic Stress in Healthcare Workers During COVID-19 Pandemic: A Review of the Immediate Impact

**DOI:** 10.3389/fpsyg.2020.569935

**Published:** 2020-10-23

**Authors:** Agata Benfante, Marialaura Di Tella, Annunziata Romeo, Lorys Castelli

**Affiliations:** Department of Psychology, University of Turin, Turin, Italy

**Keywords:** trauma, acute stress, vicarious traumatization, COVID-19, healthcare workers

## Abstract

The disease caused by respiratory syndrome coronavirus 2 (SARS-CoV-2) called COVID-19 resulted in a pandemic that has demanded extraordinary physical and mental effort from healthcare workers. This review provides an overview of studies that have explored traumatic stress in healthcare workers and associated factors between January and May 2020. The focus is on the most relevant literature investigating the prevalence of trauma‐ and stressor-related symptoms. Articles were selected from PubMed and PsycINFO databases using the search terms, “healthcare workers,” “COVID-19,” and “posttraumatic stress” in different combinations and with various synonyms. Among the seven studies that fulfilled our criteria, five assessed traumatic stress response, one assessed acute stress symptoms, and one focused on vicarious traumatization. Overall, the available findings highlight the presence of trauma-related stress, with a prevalence ranging from 7.4 to 35%, particularly among women, nurses, frontline workers, and in workers who experienced physical symptoms. Future studies should clarify the long-term effects of the COVID-19 pandemic on the mental health of healthcare workers, with particular focus on posttraumatic stress disorder.

## Introduction

The World Health Organization (WHO) declared COVID-19 as a pandemic on March 11, 2020, when infections and deaths began to increase exponentially worldwide. The first cases were reported during December 2019 in Wuhan, China (WHO, 2020).

This virus belongs to the coronavirus family, which can cause respiratory infections in humans that resemble the common cold, as well as lethal illness similar to that associated with Middle East Respiratory Syndrome (MERS) and Severe Acute Respiratory Syndrome (SARS; Carver and Phillips, 2020). The symptoms of the new respiratory syndrome coronavirus 2 (SARS-CoV-2) can be fever, cough, tiredness, pains, nasal congestion, headache, and conjunctivitis, but they can also include pneumonia, acute respiratory syndrome, kidney failure, and death. Transmission is believed to occur *via* droplets (Carver and Phillips, 2020; Lechien et al., 2020; WHO, 2020). By May 22, 2020 the number of global confirmed infections and deaths had reached ~4,893,000 and ~323,000, respectively (WHO, 2020). Such an extraordinary event will have long-term effects on mental health according to previous studies of epidemics and quarantine (Maunder et al., 2006; Brooks S. K. et al., 2020; Kisely et al., 2020). The COVID-19 pandemic is classifiable as a traumatic event of exceptional magnitude that transcends the range of normal human experience with exposure to risk of death (Dutheil et al., 2020). These aspects can trigger psychopathologies such as acute stress disorder (ASD) and posttraumatic stress disorders (PTSD). Healthcare workers (HCWs) have been faced with unprecedented demands, both professionally and personally, in efforts to manage a disease with unclear etiology and pathology, no cure, no vaccine, and a high mortality rate. They are obliged to make difficult ethical decisions and function professionally under conditions of fear for themselves and their loved ones (Dutheil et al., 2020; Gavin et al., 2020; Kisely et al., 2020; Wong et al., 2020).

The aim of this review is to provide an overview of studies focusing on traumatic stress in HCWs during the COVID-19 pandemic.

## Materials and Methods

### Research Approach

In order to determine the immediate impact of COVID-19 among HCWs in terms of stress‐ and trauma-related symptoms (TRSs), a scoping review was conducted in line with existing PRISMA guidelines. A scoping review may summarize the findings related to constructs examined with heterogeneous methods and identify the aspects that future research should focus on (Tricco et al., 2018).

### Search Strategy

**Figure 1:**
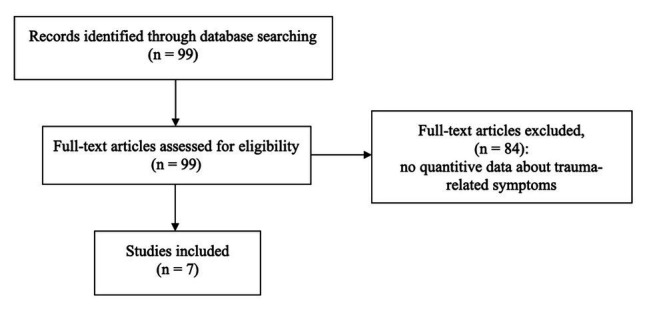
Flowchart of articles selection.

A literature search was conducted in the first 2 weeks of May 2020 in the following bibliographic databases: PubMed and PsycINFO. The databases were queried using the following strings (using Boolean operators): (“healthcare workers” OR “health care workers”) AND (“COVID-19” OR “SARS-CoV-2”) AND (“mental”); (“healthcare workers” OR “health care workers”) AND (“COVID-19” OR “SARS-CoV-2”) AND (“stress”); and (“healthcare workers” OR “health care workers”) AND (“COVID-19” OR “SARS-CoV-2”) AND (“post-traumatic stress”). The last run was conducted on May 17, 2020. With the use of this search string, 99 titles were identified between January and May 2020 (see Figure 1 for the flow diagram of article selection). Reports were also extracted using cross references, but in this way no additional article has been found.

### Eligibility Criteria

This review aimed to identify peer-reviewed academic articles that aimed to provide a measure of stress‐ or trauma-related symptoms. All types of peer-reviewed papers (original research articles, commentaries, letters to editors, and reviews) that were published in English were eligible for inclusion in this review. Exclusion criteria were as follows:

1. Papers referring to data on the impact of previous epidemics.
2. Papers that did not include validated measures to investigate stress‐ or trauma-related symptoms.
3. Studies that used *ad hoc* constructed surveys or qualitative methods.
4. Studies that included only the general population. However, the studies that used the general population as a comparison group for the HCWs have been included in the present review.
5. Papers published but not peer reviewed or under review at the time the search was carried out.

### Study Selection

Study selection was done by two authors (AB and MDT) who read the full text of all publications to screen for eligibility, because most of these articles did not include the abstracts (i.e., letter to editor and commentaries).

After the initial search, another co-author (AR) performed the literature search again, following the steps described in the study selection section above, to ensure that no records were missed and/or excluded during the selection process.

Disagreements on the inclusion or exclusion of publications were discussed by all authors until agreement was reached.

### Data Extraction

The characteristics of all included studies were extracted by one author (AB). Data items that were extracted from each included study were author and year of publication, sample and country of origin, instruments used to measure stress‐ and trauma-related symptoms and other psychological variable, trauma-related results, and other main results.

## Results of Selection

Table 1 and Appendix A summarize the main findings of the included articles. Five studies proceeded in China, one was in Singapore and one was a study of Singapore and India. All studies used self-report questionnaires disseminated through online surveys and had a cross-sectional study design. Three studies sampled only doctors and nurses, three included ancillary HCWs in hospitals such as pharmacists, physiotherapists, technicians, administrators, clerical staff, and maintenance workers, and one analyzed the general public and frontline and non-frontline nurses (nFLNs). All studies found higher percentages of females (64.3–82.7%) and nurses (up to 82.7%), with average ages ranging from 26 to 40 years (Chew et al., 2020; Kang et al., 2020; Lai et al., 2020; Li et al., 2020; Tan et al., 2020; Xiao et al., 2020; Zhang et al., 2020).

**Table 1:** Summary of trauma-related results of the included studies.

Authors	Samples (n) – location	Instrument	Trauma-related results
Chew et al. (2020)	HCWs (906) – Singapore (480) and India (426)	IES-R^†^	7.4% (67) exceeded cut-off for TRSs; 7.5 and 7.3% of HCWs from Singapore and India, respectively. Thirty-four total respondents had moderate to severe symptoms. People with physical symptoms were more likely to screen positive.
Kang et al. (2020)	Medical staff (994): doctors (183) and nurses (811) – China	IES-R^*^	Mean (SD) IES-R scores are 6.1 (4.4.), 22.9 (4.8), 39.9 (5.4), and 60 (9.8) in groups with subthreshold, mild, moderate, and severe mental disturbance, respectively. Exposure to infected persons increased for each group.
Lai et al. (2020)	Medical staff (1257): doctors (493) and nurses (764) – China	IES-R^*^	71.5% (899) had TRSs; levels were moderate to severe in 35% (440): 163 (33%) physicians and 277 (36.2%) nurses. Being female, intermediate professional titles and frontline work were associated with severe TRSs; working outside Hubei province was associated with lower risk of TRSs.
Li et al. (2020)	FLNs (234), nFLNs (292), and general public (214) – China	Vicarious traumatization questionnaire	Scores were significantly lower for FLNs than general public and nFLNs. No significant difference was found between general public and nFLNs. nFLNs had significantly increase scores than FLNs.
Tan et al. (2020)	HCWs (470) Singapore	IES-R^†^	7.7% (36) screened positive for TRSs. IES-R scores were significantly higher for non-medical, than medical staff with means (SD) of 9.4 (10.1) and 5.8 (9.2), respectively.
Xiao et al. (2020)	Medical staff (180): doctors (82) and nurses (98) – China	SASR	Mean (SD) SASR score was 77.6 (29.5). Social support and self-efficacy scores were negatively correlated with stress scores; anxiety scores were positively correlated with stress scores; SARS scores were positive correlated with sleep quality scores.
Zhang et al. (2020)	HCWs (1563) – China	IES-R^*^	73.4% had TRSs. Comparisons on impact of event between individuals with and without insomnia: sub-clinical (3.4 vs. 39.7%), mild (23.9 vs. 42.7%), moderate (42.7 vs. 15.8%), and severe (30 vs. 1.7%) TRSs.

FLNs, frontline nurses; HCWs, healthcare workers; IES-R, Impact of Event Scale – Revised; nFLNs, non-frontline nurses; SASR, Stanford Acute Stress Reaction questionnaire; SD, standard deviation; and TRSs, trauma-related symptoms.

* Cut-off > 26. Scores: normal/sub-clinical (0–8), mild (9–25), moderate (26–43), and severe distress (44–88).

† Cut-off > 24 for clinical relevance of trauma-related symptoms. Scores: normal (0–23), mild (24–32), moderate (33–36), and severe (>37).

The studies examined acute stress reaction (*n* = 1), vicarious traumatization (*n* = 1), and traumatic stress (*n* = 5). Their findings are discussed below.

### Acute Stress Reaction

Xiao et al. (2020) investigated acute stress response among medical staff. Acute stress reaction is an anxious response, which in its most serious cases can be accompanied by manifestations associated with reliving the traumatic event or signs of reactivity (Walton et al., 2020). In accordance with the criteria of fifth edition of the Diagnostic and Statistical Manual of Mental Disorders (DSM-5), a diagnosis of ASD requires at least nine of 14 symptoms, including negative mood, intrusion, dissociation, avoidance, and arousal (such as sleep difficulties, irritability, and inattention), that were initiated or worsened shortly after the event (Bryant, 2018). The study of Xiao et al. (2020) was conducted during the 1st month of the COVID-19 outbreak in China and the Stanford Acute Stress Reaction (SASR) questionnaire was used. This questionnaire evaluates consequential symptoms of traumatic events with higher scores corresponding to higher levels of stress-related symptomatology (range 0–150; Cardeña et al., 2000). The average score of for SASR was 77.6. Social support and self-efficacy scores correlated negatively with stress scores, and positive correlations were identified between anxiety and stress scores and between stress and sleep quality scores in that study. The main objective of that study was to determine the effects of social support on sleep quality among doctors and nurses, considering several other psychological aspects. Associated with this, social support indirectly affected the sleep quality of HCWs, reduced stress and anxiety levels and improved self-efficacy, while confirming that high levels of stress (with high anxiety levels and low self-efficacy) reduce sleep quality (Xiao et al., 2020).

### Vicarious Traumatization

Li et al. (2020) investigated levels of vicarious traumatization in frontline and non-frontline nurses and in a general population. The concept of vicarious traumatization, also defined as secondary traumatic stress, includes various traumatic conditions, in which psychological abnormalities are related to the sympathy of HCWs toward people who are primarily traumatized. The symptoms associated with vicarious traumatization are loss of appetite, fatigue, sleep disorders, irritability, inattention, fear, and interpersonal conflict, which often remain at sub-clinical levels (Sabin-Farrell and Turpin, 2003; Li et al., 2020). The questionnaire adopted in their study comprised physiological and psychological dimensions. The psychological dimension included items associated with emotional, behavioral, and cognitive responses, and life beliefs. The results suggested that the general public, frontline and non-frontline nurses suffered from vicarious traumatization, but between-group differences emerged. Frontline nurses (FLNs) had significantly lower scores than the other two groups, which did not significantly differ. In addition, married, divorced, or widowed nurses had more severe symptoms than unmarried nurses.

These results might be explained by the fact that the frontline nurses were composed of voluntarily selected professionals, who were trained with sufficient psychological preparation, with a middle-level professional title, and with work experience. Furthermore, the increased vicarious traumatization of nFLNs, as well as of general public, would derive from the sympathy and worry felt for COVID-19 patients and frontline workers, who instead sympathize only with patients and are more experienced about pandemic (Li et al., 2020).

Considering the recognition of the propensity of frontline nurses to suffer from vicarious traumatization (Taylor et al., 2016), it is essential to pay attention to the psychological health of these professionals, but also to take care of nFLNs, according to the findings of the study of Li et al. (2020).

### Traumatic Stress

Five selected studies investigated the psychological impact of COVID-19-related trauma in HCWs using the Impact of Event Scale – Revised (IES-R; Chew et al., 2020; Kang et al., 2020; Lai et al., 2020; Tan et al., 2020; Zhang et al., 2020). The IES-R is a 22-item scale (range 0–88) that measures intrusive, avoidance, and hyperarousal symptoms typical of trauma. It is relatively independent from trauma‐ and stress-related disorders included in DSM-5, but considering that it examines symptoms in PTSD, it has often been used to identify this disorder (Wu and Chan, 2003). Chinese studies interpreted the IES-R scores as follows: normal/sub-clinical (0–8), mild (9–25), moderate (26–43), and severe distress (44–88), with a cut-off of 26 (Wu and Chan, 2003; Kang et al., 2020; Lai et al., 2020; Zhang et al., 2020). In contrast, studies conducted in Singapore and India evaluated IES-R scores as follows: normal (0–23), mild (23–32), moderate (33–36), and severe (>37), with a cut-off of 24 indicating possible PTSD (Creamer et al., 2003; Chew et al., 2020; Tan et al., 2020). These studies included 470–1,563 respondents (Tan et al., 2020; Zhang et al., 2020).

Lai et al. (2020) conducted a hospital-based survey that was stratified for the region where the participants worked. The IES-R scores showed that 899 (71.5%) of 1,257 physicians and nurses had traumatic stress symptoms and the level was moderate/severe in 440 (35%) of them. Specifically, 163 (33%) physicians and 277 (36.2%) nurses had clinically relevant symptoms (Lai et al., 2020).

Furthermore, women, nurses, and those working in Wuhan reported more severe symptoms of trauma stress and worse outcomes for anxiety, depression, and insomnia, with respect to men, physicians, and those working in Hubei outside Wuhan and outside Hubei. In particular, being women and having an intermediate technical title were associated with increased anxiety, depression, and TRSs. Being a frontline worker, directly engaged in the diagnosis and treatment of patients infected with COVID-19, was an independent risk factor for higher scores not only at the IES-R, but also at other measures used in the study (Lai et al., 2020). These prevalences were similar to those of Zhang et al. (2020), who found that 73.4% of HCWs respondents had IES-R scores ≥9, indicating the presence of traumatic stress symptoms. The main objective of that study was to determine the prevalence of insomnia and associated factors, the authors compared the levels of psychological impact of the event between HCWs with and without insomnia. Individuals with insomnia reported a significantly higher psychological impact compared to those without insomnia (symptoms of traumatic stress based on IES-R: moderate 42.7 vs. 15.8% and severe 30 vs. 1.7%; Zhang et al., 2020).

Kang et al. (2020) conducted a study on the mental health of HCWs, exploring also their psychological needs and access to mental health services. The HCWs were assigned to four groups (1–4) based on scores for depression, anxiety, insomnia, and traumatic stress. Groups 1, 2, 3, and 4 had subthreshold, mild, moderate, and severe disturbances (36, 34, 22.4, and 6.2% of the sample, respectively) and mean IES-R scores of 6.1, 22.9, 39.9, and 60, respectively. These findings indicated that group 4 was exposed to possible COVID-19 positive persons more often, had less access to psychological material and worse self-perceived health status, than the other three groups. Ultimately, that study showed that exposure to infected patients negatively impacted mental health, which in turn influenced subjective perception of physical health. Access to mental health services had a partial mediating effect between the risk of contact with COVID-19 positive patients and the mental health of the respondents (Kang et al., 2020).

The prevalence data differed in other countries. Tan et al. (2020) found that only 7.7% of their respondents screened positive for TRSs. In addition, the percentage was higher among non-medical, than medical personnel (10.9 vs. 5.7%). The authors assumed that the scores were lower than those found in studies of previous epidemics because the medical personnel might have been more mentally prepared due to previous experience (Tan et al., 2020). The results of the study by Chew et al. (2020) were similar; 7.4% of the total sample of HCWs exceeded the IES-R cut-off (Singapore, 7.5%; India, 7.3). Like to the finding of Chew et al. (2020) and Kang et al. (2020) associated having physical symptoms with an increased probability of high scores for trauma-related stress. A possible explanation for this result is that nonspecific symptoms, such as headache, sore throat, cough, breathlessness, lethargy, myalgia, and loss of appetite, are also part of the symptomatology of milder forms of COVID-19 infection (Chew et al., 2020; Lechien et al., 2020).

Thus, the presence of TRSs differed according to IES-R in these studies, with prevalence ranging from 7.4 to 35% (Chew et al., 2020; Lai et al., 2020).

## Discussion

To the best of our knowledge, this is the first review on the issue of COVID-19 trauma‐ and stress-related symptoms in HCWs. Other literature reviews of previous epidemics and/or the COVID-19 pandemic have focused on generic psychological distress and/or anxiety and depressive symptoms. Meta-analyses have found a high prevalence of anxious and depressive symptoms among HCWs, especially among women and nurses (Pan et al., 2020; Pappa et al., 2020). In addition, a series of recent reviews highlighted that risk factors, such as being female, younger, being a nurse, lack of adequate protective equipment, and exposure to infected people, have been found to be associated to TRSs in previous epidemics (Brooks S. K. et al., 2020; Kisely et al., 2020; Rajkumar, 2020; Spoorthy, 2020; Walton et al., 2020).

Regarding the recent COVID-19 outbreak, the available studies show an important presence of COVID-19 trauma and stress-related symptoms in the general population and in patients (Bo et al., 2020; Rajkumar, 2020; Ren et al., 2020; Wang et al., 2020). However, to date, only few studies have analyzed this specific aspect in HCWs.

The psychological traumatic impact of COVID-19 in frontline and non-frontline HCWs is a great issue, as emerged by almost all the included studies (Kang et al., 2020; Lai et al., 2020; Xiao et al., 2020; Zhang et al., 2020). Contrasting results seem to emerge only in the studies of Lai et al. (2020) and Li et al. (2020), which found a different prevalence of TRSs between frontline vs. non-frontline HCWs. However, this discrepancy could be explained considering the different constructs the two studies examined and the heterogeneity of the samples they enrolled.

The present review highlighted an important impact of the COVID-19 pandemic on the mental health of HCWs. The prevalence of clinically relevant TRSs ranged from 7.4 to 35% (Chew et al., 2020; Lai et al., 2020), while in Chinese general population the prevalence of TRSs is ~7% (Ren et al., 2020; Wang et al., 2020). The differences among these results could be explained by different contagion rates and pressure on health care systems, the different incidence of the risk factors and different of access to psychological support. Particularly, being female, younger, a frontline worker, a nurse, having less work experience, exposure to infected people, poor social support, difficult access to psychological material, insomnia and physical symptoms are all risk factors for traumatic symptoms in HCWs (Chew et al., 2020; Kang et al., 2020; Lai et al., 2020; Xiao et al., 2020).

Furthermore, the multiple sources of distress that face HCWs are important to consider, such as concern about the spread of the virus, their own health, the health of their loved ones, and changes in the work environment (Cacchione, 2020; Gavin et al., 2020; Lai et al., 2020; Menon and Padhy, 2020; Neto et al., 2020). The HCWs are also at risk for moral injury, that is psychological distress derived from actions (or the impossibility of implementing actions) that violate their personal ethical and moral codes (Greenberg et al., 2020; Williamson et al., 2020). All these aspects contribute to the possibility that HCWs develop psychopathological disorders such as PTSD, severe depression, and substance abuse (Brooks S. K. et al., 2020). Future studies should clarify the long-term effects of the COVID-19 pandemic on the mental health of HCWs, with particular focus on PTSD.

However, HCWs that appear to be less at risk or who have mild traumatic stress symptoms should also be considered (Chew et al., 2020; Kang et al., 2020; Li et al., 2020; Tan et al., 2020). For example, Kang et al. (2020) showed that HCWs with low levels of mental health disturbances expressed the need to improve their skills to mitigate mental distress, both for themselves and for others.

Early symptoms of psychological trauma, together with symptoms of anxiety, depression, and insomnia, must be recognized, so that appropriate interventions can consider the organizational needs of HCWs, risk and protective factors, and possibly include actions to promote post-traumatic growth (Brooks S. et al., 2020; Conversano et al., 2020; Romeo et al., 2020; Shah et al., 2020; Shanafelt et al., 2020). The literature suggests that people exposed to trauma can experiment with positive responses, reconsidering their values and appreciating their lives more as well as their work in emergency situations. These aspects can be fostered by psychological interventions (Xu et al., 2016; Brooks S. et al., 2020).

This review has some limitations, due both to the limited number of studies specifically investigating post-traumatic symptoms of COVID-19 on HCWs, and to the methodological differences (e.g., cross-sectional design) of the selected studies themselves.

## Author Contributions

AB, MDT, AR, and LC conceived and designed the review. AB, MDT, and AR carried out the literature searches and screening. AB and LC wrote the manuscript. All authors concluded the results, discussed and approved the final version of the manuscript.

### Conflict of Interest

The authors declare that the research was conducted in the absence of any commercial or financial relationships that could be construed as a potential conflict of interest.
