# Supplementary material for: Traumatic Stress in Healthcare Workers During COVID-19 Pandemic: A Review of the Immediate Impact
Source: Front Psychol. 2020 Oct 23;11:569935. doi: 10.3389/fpsyg.2020.569935 (PMC7645025; doi:10.3389/fpsyg.2020.569935)
Supplement: Supplementary file 1 [file Table_1.docx]

**Appendix A.** Summary of the main findings related to the other variables examined in the studies.

| Authors | Samples (n) – Location | Other instruments | Other main results |
| --- | --- | --- | --- |
| Chew et al., 2020 | HCWs (906) – Singapore (480), India (426) | DASS-21 | 15.7% (142), 10.6% (96) and 5.2% (47) exceeded cut-off for anxiety, depression and stress, respectively; no difference emerged between the HCWs from the two countries.  People with physical symptoms (respiratory-related and constitutional symptoms) were more likely to screen positive for all psychological outcomes. |
| Kang et al., 2020 | Medical staff (994): doctors (183),  nurses (811) – China | PHQ-9, GAD-7, ISI | 36% (mean ± SD: PHQ-9: 2.4 ± 3.0, GAD-7: 1.5 ± 2.4, ISI: 6.1 ± 4.0), 34.4% (mean ± SD: PHQ-9: 5.4 ± 3.4, GAD-7: 4.6 ± 2.9, ISI: 6 ± 4.0), 22.4% (mean ± SD: PHQ-9: 9.0 ± 3.9, GAD-7: 8.2 ± 3.6, ISI: 10.4 ± 4.8) and 6.2% (mean ± SD: PHQ-9: 15.1 ± 5.2, GAD-7: 15.1 ± 4.3, ISI: 15.6 ± 5.2) had subthreshold, mild, moderate and severe mental disturbances, respectively  Exposure to infected persons increased for each group. Those with severe mental disturbances had access to less psychological materials and resources. Current health status perception, compared to the one before, was different among the groups.  Exposure to infected patients affected mental health, with partially mediation of mental services. Mental health affected physical health perception. Mental services regulated relationship between exposure and physical health perception by affecting mental health.  Staff with subthreshold disturbances wanted to obtain skills to alleviate others’ psychological distress, the remaining wanted to obtain self-help competence. |
| Lai et al., 2020 | Medical staff (1257): doctors (493), nurses(764) – China | PHQ-9, GAD-7, ISI | 50.4% (634), 44.6% (560) and 34.0% (427) had symptoms of depression, anxiety and insomnia, respectively.  Nurses, women, frontline workers and those in Wuhan experienced more severe psychological symptoms. Workers in secondary hospitals workers were more likely to experience symptoms of depression, anxiety, and insomnia. Similar results emerged for median scores in all scales used.  Being female and having intermediate professional titles were associated with severe symptoms of depression and anxiety. Working in frontline seems to be an independent risk factor for all symptoms. |
| Tan et al., 2020 | HCWs (470)  Singapore | DASS-21 | 14.5% (68), 8.9% (42) and 6.6% (31) for anxiety, depression and stress, respectively. Anxiety and stress scores were significantly higher for non-medical, than medical staff with means (SD) of 3.6 (3.9) vs. 2.5 (4.3) and 6.1 (5.9) vs 3.3 (5.7), respectively.  Anxiety prevalence was higher among non-medical than medical HCWs. |
| Xiao et al., 2020 | Medical staff (180): doctors (82),  nurses (98) – China | SSRS, SAS, GSES, PSQI | Means (SD) of social support, self-efficacy, anxiety and sleep quality were 34.2 (10.3), 2.3 (0.8), 55.3 (14.2) and 8.6 (4.6), respectively.  There was significantly positive correlation between scores in: SSRS and GSES; SAS and PSQI. There was significantly negative correlation between scores in: SSRS and SAS; SSRS and PSQI; GSES and SAS; GSES and PSQI.  SSRS scores negatively affected the SAS scores; SAS scores positively affected PSQI scores, and negatively affected GSES scores. GSES score negatively affected the PSQI scores. Effect of SSRS scores on PSQI scores was not significant. |
| Zhang et al., 2020 | HCWs (1563) – China | ISI, PHQ-9, GAD-7 | 36.1% had symptoms of insomnia. Prevalence of symptoms of depression and anxiety were 50.7% and 44.7%, respectively.  Several sociodemographic and COVID-19-related factors were found to be involved in a greater or lesser probability of insomnia symptoms.  Comparisons on depressive and anxiety symptoms between individuals with and without insomnia: minimal/no (14.5% vs. 69% and 19.3% vs.75.6%), mild (45.9% vs 26.4% and 50.7% vs 21.1%), moderate (22.9% vs 2.8% and 18.3% vs 1.6%), and severe levels (16.7% vs 1.8% and 11.7% vs 1.7%).  Having a low-medium level of education, being a doctor, working in isolation units, concern about being infected, perceived psychological support not useful, and very strong uncertainty about effective control of the disease were statistically significant predictors of insomnia. |

Note: DASS-21, Depression Anxiety Stress Scale; FLNs, frontline nurses; GAD-7, Generalized Anxiety Disorder; GSES, General Self-Efficacy Scale; HCWs, healthcare workers; ISI, Insomnia Symptoms Index; nFLNs, non-frontline nurses; PHQ-9, Patient Health Questionnaire; PSQI, Pittsburgh Sleep Quality Index; SAS, Self-rating Anxiety Scale; SD, standard deviation; SSRS, Social Support Rate Scale.
